# Mitral Cell Dendritic Morphology in the Adult Zebrafish Olfactory Bulb following Growth, Injury and Recovery

**DOI:** 10.3390/ijms25095030

**Published:** 2024-05-05

**Authors:** John P. Rozofsky, Joanna M. Pozzuto, Christine A. Byrd-Jacobs

**Affiliations:** 1 Department of Biological Sciences, Western Michigan University, 1903 W Michigan Ave., Kalamazoo, MI 49009, USA; john.p.rozofsky@wmich.edu; 2 Department of Biology, Kalamazoo Valley Community College, 6767 W O Ave., Kalamazoo, MI 49009, USA; jpozzuto@kvcc.edu

**Keywords:** zebrafish, deafferentation, olfactory bulb, mitral cell, retrograde labeling

## Abstract

The role of afferent target interactions in dendritic plasticity within the adult brain remains poorly understood. There is a paucity of data regarding the effects of deafferentation and subsequent dendritic recovery in adult brain structures. Moreover, although adult zebrafish demonstrate ongoing growth, investigations into the impact of growth on mitral cell (MC) dendritic arbor structure and complexity are lacking. Leveraging the regenerative capabilities of the zebrafish olfactory system, we conducted a comprehensive study to address these gaps. Employing an eight-week reversible deafferentation injury model followed by retrograde labeling, we observed substantial morphological alterations in MC dendrites. Our hypothesis posited that cessation of injury would facilitate recovery of MC dendritic arbor structure and complexity, potentially influenced by growth dynamics. Statistical analyses revealed significant changes in MC dendritic morphology following growth and recovery periods, indicating that MC total dendritic branch length retained significance after 8 weeks of deafferentation injury when normalized to individual fish physical characteristics. This suggests that regeneration of branch length could potentially function relatively independently of growth-related changes. These findings underscore the remarkable plasticity of adult dendritic arbor structures in a sophisticated model organism and highlight the efficacy of zebrafish as a vital implement for studying neuroregenerative processes.

## 1. Introduction

The zebrafish olfactory system provides an excellent platform for the study of brain plasticity due to the well-known regenerative abilities of zebrafish [1] and the inherent constitutive and regenerative processes of the olfactory system [2,3]. As in other animals, zebrafish exhibit life-long plasticity in both the olfactory epithelium and the olfactory bulb [4,5]. Several methods have been developed in adult zebrafish to cause damage to the olfactory organ that results in deafferentation of the olfactory bulb [6,7,8,9,10]. Degeneration and regeneration of the zebrafish olfactory bulb following deafferentation has been shown to result in numerous changes to bulb morphology and neurochemistry [6,7]. These effects are accompanied by social behavioral deficits that return to normal when given time to recover [8,11]. Of note for the current study is the ability to perform repeated chemical ablation to the olfactory epithelium to cause long-term, chronic, partial deafferentation of the olfactory bulb [7], resulting in morphological, pharmacological, and behavioral effects. With cessation of treatment, the olfactory bulb recovers, showing that this is a reversible deafferentation model [11]. Calvo-Ochoa and Byrd-Jacobs [5] performed an extensive review of deafferentation-induced changes in the zebrafish olfactory bulb that outlines additional results and reinforces the use of zebrafish as a model system for neural plasticity.

The teleost olfactory bulb is organized in a diffusely ordered laminar structure, with output neurons found throughout the glomerular layer and mitral cell (MC) dendrites synapsing with olfactory sensory neuron axons [12]. Zebrafish MCs are morphologically heterogeneous and possess both multi- and uni-dendritic arbors [12]. Upon receiving sensory information from the olfactory epithelium, MCs serve as the primary relay neurons within the olfactory bulb propagating the signal from the olfactory bulb to higher order brain structures via the medial and lateral olfactory tracts [13,14,15]. The role of afferent-target interactions in maintenance of dendritic morphology in the adult brain is not well understood, and a reversible deafferentation technique is effective in the exploration of this process.

Dendritic trees form a foundational network in the nervous system, and plasticity of dendritic structures relates to injury and recovery processes within the adult brain. Numerous animal models have been used to study dendritic plasticity. In mice, exposure to physical and social stress is associated with increased dendritic spine density in the nucleus accumbens, with adolescents showing different responses to social stress compared to adults [16]. Additionally in mice, whisker trimming induces functional reorganization of intracortical circuits within the barrel cortex resulting in destabilization of persistent spines and stabilization of new spines in pyramidal neurons [17]. Dendrites of optic tectal neurons in young *Xenopus* substantially increase in complexity when exposed to tyrosine hydroxylase [18]. It is rarer to find studies of dendrite plasticity in adult animals, but zebrafish provide a good model for this. For example, in the retina of adult zebrafish, dendritic field size and complexity of bipolar neurons is restored after chemical damage, showing recovery of dendritic morphology in an adult [19].

The olfactory system’s inherent adaptability makes it a good model for this area of study. In the *Xenopus* olfactory bulb, dendritic plasticity is influenced by olfactory stimulation and deprivation. Single-cell electroporation of granule cells with green fluorescent protein allowed for short and long-term imaging of synaptic structure plasticity with odor stimulation resulting in increased stability of dendrites [20]. Another study in *Xenopus* used sparse cell electroporation of a dextran to show that mitral/tufted cell dendritic tufts are less complex and smaller following olfactory nerve transection [21]. This deafferentation effect is transient and dendritic complexity returns with reinnervation. In rodents, large scale plasticity of dendritic dynamics has been shown in numerous cell types in the olfactory bulb. Periglomerular neurons in the mouse olfactory bulb imaged using two photon microscopy under odor-enriched environments demonstrate accelerated dendritic development [22]. Adult-born mouse granule cell dendrites are regulated in early maturational stages through the activation of NMDA receptors [23]. Information specific to zebrafish dendritic plasticity is lacking, despite that model organism’s prevalence in regeneration studies.

MC dendritic morphology is impacted by loss of afferent input. Permanent ablation of the olfactory epithelium in adult zebrafish disrupts glomerular structures after 3 weeks, with fluorescently labeled phallotoxin showing loss of axonal and dendritic components [24]. Following 8 weeks of permanent deafferentation, phallotoxin labeling shows indistinguishable glomeruli and decreased bulb volume. In addition, retrograde labeling of MCs reveals significant reductions in total length of major dendritic branches, area of the dendritic field, and optical density measures of the fine processes in the dendritic tuft [24]. Similarly, 8 weeks of chronic, partial deafferentation results in numerous morphological changes in MC dendrites and significant reductions in olfactory bulb MC dendritic arborization measures including modified Sholl analysis, number of major branches, total length of major dendritic branches, area of dendritic field, and distribution of fine processes [24]. Given that this chronic, partial deafferentation method in zebrafish is reversible, it is possible to explore the ability of MC dendrites to recover from this deafferentation-induced damage.

Building upon the results of Pozzuto and colleagues [24] demonstrating the loss of MC dendritic structures following injury in zebrafish, this study examined the effects of cessation of treatment after long-term chronic deafferentation in order to explore dendritic plasticity during recovery from damage. Our hypothesis was that the cessation of deafferentation would allow for the recovery of MC dendritic arbor structure and complexity. Additionally, considering that zebrafish exhibit continued growth with age [25] and these experiments included survival times of up to four months, we hypothesized that there would be changes in dendritic arbor structures due to growth.

## 2. Results

### 2.1. Recovery of Mitral Cell Dendritic Arbors

**Figure 1:**
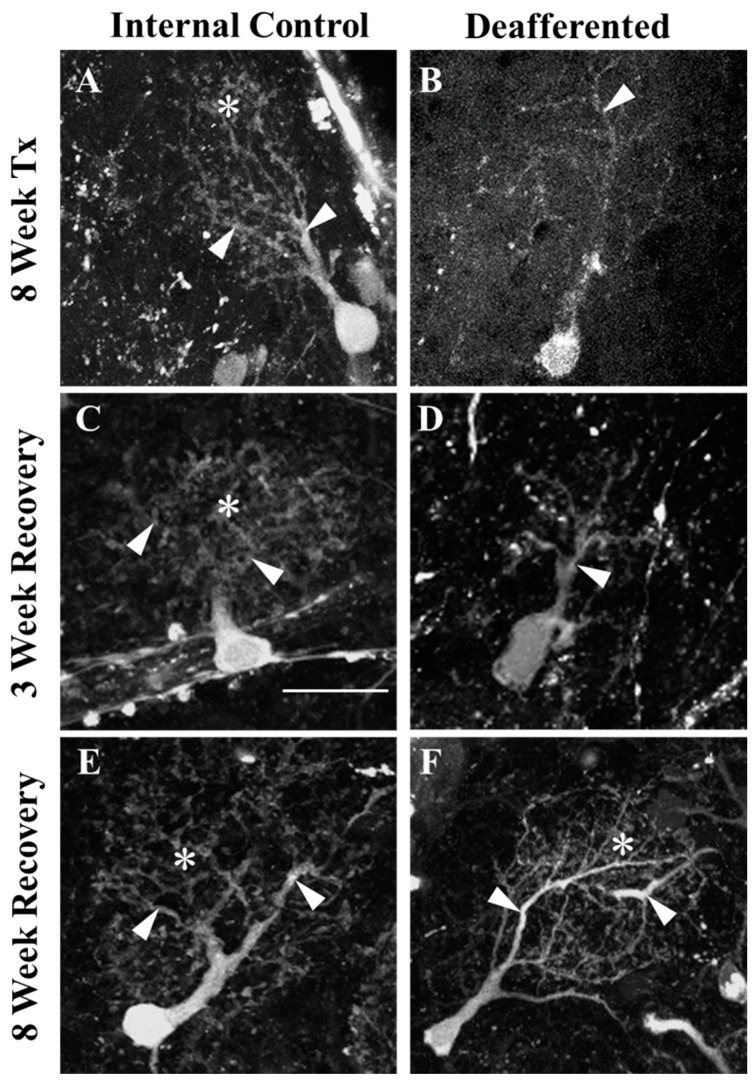
The ability of mitral cell dendritic arbor morphology to recover was examined at 3 and 8 weeks after cessation of treatment following 8 weeks of chronic, partial deafferentation. (**A**) Control mitral cell dendritic arbors had numerous branches (arrowheads) and a dense tuft of fine processes (*). With deafferentation (**B**), there was an obvious reduction in fine processes and branches. When allowed 3 weeks of recovery, internal control mitral cells (**C**) exhibited control morphologies. Previously deafferented mitral cells (**D**) still lacked smaller branches and fine processes typical of control cells, though major branches were present (arrowheads) and were more abundant than in deafferented cells. Internal control cells (**E**) following 8 weeks of recovery exhibited control morphologies and appeared to be larger in size than control cells at previous time points. Following 8 weeks of chronic partial deafferentation and 8 weeks of recovery, mitral cell dendritic arbors appeared to return to control morphology, with abundant primary, major, and fine dendritic branches (**F**). Scale = 20 μm, for all.

Our previous study showed that 8 weeks of repeated chemical ablation of the olfactory epithelium causes significant effects on MC dendritic arbors (Figure 1A,B), including diminished secondary branches, reduction in the number of major dendritic branches, significantly reduced total length of major dendritic branches, decreased relative size of dendritic arbor, and reduction in the optical density of the distribution of fine processes within the dendritic arbor [24]. To explore the ability of MC dendrites to recover from these deafferentation-induced effects, dendritic arbors were analyzed for complexity after various time periods following reinnervation, using our reversible deafferentation model [7]. Morphological analysis included the previously mentioned measures, as well as modified Sholl analysis, performed 3 and 8 weeks after cessation of treatment (Figure 1C–F).

**Figure 2:**
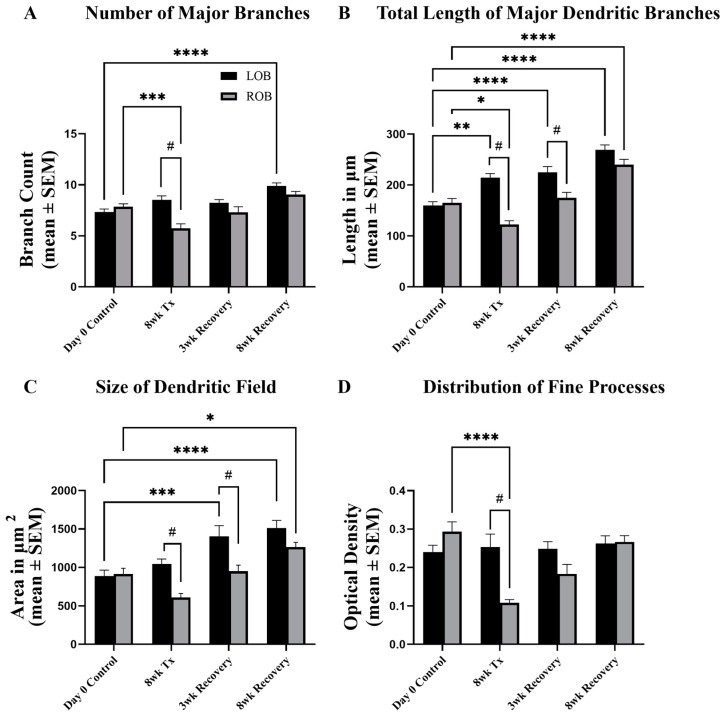
The potential of mitral cell dendritic arbor structures to recover following chronic, partial deafferentation was investigated. (**A**) Following 8 weeks of chronic, partial deafferentation there was a significant reduction in the number of major branches compared to internal control and unlesioned day 0 control cells. With 8 weeks of recovery there were significantly more major branches in internal control bulbs compared to day 0 controls. (**B**) Eight weeks of chronic, partial deafferentation results in effects on total length of mitral cell major dendritic branches. With 3 weeks of recovery, significant decreases remained, while the length of major dendritic branches within the internal control bulb were still significantly greater than those in day 0 unlesioned control bulbs. Following 8 weeks of recovery there was a significant increase compared to day 0 control cells, but no differences were seen between internal control and treated sides. (**C**) There was a significant decrease in the relative size of the dendritic field following 8 weeks of partial chronic deafferentation and these effects remained significantly reduced when allowed 3 weeks of recovery. There were significant increases in internal control cells compared to day 0 unlesioned control cells with 3 weeks of recovery, and in both internal control and previously deafferented cells following 8 weeks of recovery. (**D**) Following 8 weeks of partial chronic deafferentation there was a significant decrease in optical density of the dendritic arbor compared to day 0 unlesioned control cells, and there was a difference between internal control and treated sides. LOB = left, internal-control olfactory bulb, ROB = right, treated olfactory bulb. For day 0 control, n = 5 fish, 26 mitral cells (LOB), 28 mitral cells (ROB). For 8wk TX, n = 4 fish, 21 mitral cells (LOB), 26 mitral cells (ROB). For 3wk Rec, n = 4 fish, 21 mitral cells (LOB), 20 mitral cells (ROB). For 8wk Rec, n = 5 fish, 26 mitral cells (LOB), 26 mitral cells (ROB). Paired t-test compared to internal controls, # = *p* < 0.05; Two-way ANOVA, with Tukey’s multiple comparisons test compared to day zero controls, **** = *p* < 0.0001; *** = *p* < 0.001; ** = *p* < 0.01; * = *p* < 0.05. F-value is 16.31 (**A**), 44.22 (**B**), 19.75 (**C**), and 7.58 (**D**). Degrees of freedom is three, for all. 95% Confidence Interval is 0.51 to 1.53 (**A**), 28.66 to 54.65 (**B**), 158.90 to 392.90 (**C**), and 0.01 to 0.07 (**D**).

There was a significant reduction in the number of major branches in MC dendritic arbors at 8 weeks post-deafferentation as reported previously [24]. When allowed 3 weeks of recovery, the number of major branches of previously deafferented cells returned to control levels, with no significant differences when compared to internal control cells and unlesioned control cells (Figure 2A). Additionally, following 8 weeks of recovery the total number of major branches within MC dendritic arbors in internal control cells was increased significantly compared to unlesioned control cells (Figure 2A).

It was previously shown that following 8 weeks of chronic, partial deafferentation there is a significant decrease in the length of major branches in deafferented cells compared to internal control and unlesioned control cells, and cells in the contralateral internal control bulb at 8 weeks post-deafferentation are significantly longer than those in unlesioned control cells [26]. At 3 weeks following cessation of deafferentation there were significant decreases in the total length of major dendritic branches when compared to internal control cells (Figure 2B). Furthermore, the total length of major dendritic branches of internal control MCs was significantly increased from the lengths of those in unlesioned control fish. With 8 weeks of recovery there were no significant differences in major dendritic branch length between the left and right olfactory bulbs of previously deafferented animals; however, the total dendritic branch lengths of these cells were significantly longer than those of the unlesioned control fish quantified at day 0 (Figure 2B).

Projections of z-stack images were used to estimate the relative size of the dendritic arbor. At 8 weeks post-deafferentation there is a significant decrease in the size of the dendritic field between dendritic arbors of deafferented cells compared to internal control cells and unlesioned control cells, as shown before [24]. With 3 weeks of recovery, there were significant decreases in MC dendritic arbor size between the left and right olfactory bulbs, and the size of the dendritic arbors in the internal control bulb were significantly increased compared to unlesioned control animals (Figure 2C). Following 8 weeks of recovery, MC dendritic arbor size was significantly larger in both the left and right olfactory bulbs compared to unlesioned control cells (Figure 2C).

The optical density for dextran labeling of the dendritic arbor was used to estimate the effects of deafferentation on the fine processes of the dendritic arbor that could not be accurately traced. Pozzuto et al. [24] showed previously that with 8 weeks of partial chronic deafferentation there is a significant decrease in the optical density of dextran labeling in deafferented MC dendritic arbors compared to internal control cells and unlesioned control cells. When allowed 3 and 8 weeks of recovery there were no significant differences in the distribution of fine processes of the dendritic arbor between left and right olfactory bulbs of recovered animals (Figure 2D).

**Figure 3:**
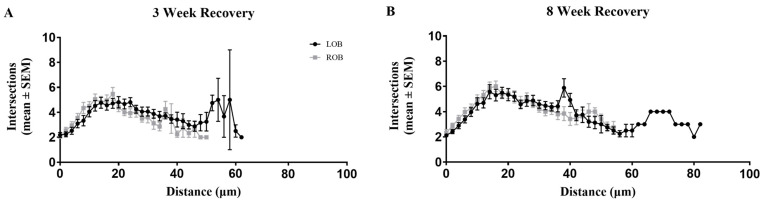
A modified Sholl analysis was used to determine whether the significant decline in overall dendritic complexity that occurs following 8 weeks of chronic, partial deafferentation could be reversed. (**A**) When allowed to recover for 3 weeks, there were no significant differences (*p* = 0.63) in the number of intersections at any distance from the base of the arbor or in overall dendritic complexity, though there is some variability: n = 4 fish, 21 mitral cells (LOB), 20 mitral cells (ROB). (**B**) Following 8 weeks of recovery there were no significant differences (*p* = 0.08) in the number of intersections or in overall dendritic complexity: n = 5 fish, 26 mitral cells (LOB), 26 mitral cells (ROB). Kolmogorov-Smirnov test compared lines formed by the average number of intersections at various distances from the base of the arbor. LOB = left, internal-control olfactory bulb, ROB = right, treated olfactory bulb.

Modified Sholl analyses were used to estimate the recovery of dendritic arbor complexity following deafferentation. Due to dendritic arbor distance from the soma in unidendritic zebrafish MCs, a modified Sholl analysis where the concentric circles started at the base of the arbor was used to examine the overall complexity of the dendritic arbors [24]. Our previous study showed that 8 weeks of partial, chronic deafferentation resulted in a significant decrease in the overall complexity of MC dendritic arbors [24]. Following 3 and 8 weeks of recovery, overall dendritic complexity returned to control levels, with no significant differences in the complexity of MC dendritic arbors between left and right olfactory bulbs of previously deafferented animals (Figure 3A,B).

### 2.2. Growth

**Figure 4:**
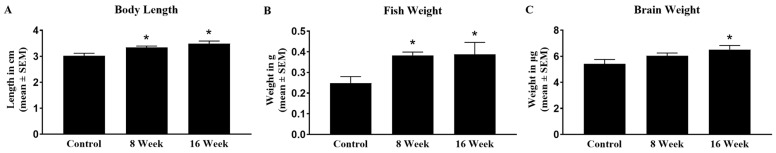
The potential growth of unlesioned control animals over 16 weeks was examined. (**A**) There was a significant increase in body length compared to day 0 control animals (n = 5) at both 8 (n = 4) and 16 (n = 4) weeks. (**B**) After 8 and 16 weeks there were significant increases in animal weight compared to day 0 control animal weights. (**C**) Brain weights of unlesioned cohort control animals at 16 weeks were significantly increased compared to day 0 control animals. Unpaired t-test, * = *p* < 0.05 compared to day 0 unlesioned control animals.

Examination of the above data led to the observation that 8 weeks of deafferentation and 8 weeks of recovery, totaling 16 weeks after unlesioned control fish were processed, appeared to allow substantial growth of dendritic arbor structures. To examine the effects of potential growth-related changes to the dendritic arbors of zebrafish MCs over time, a group of cohort control animals were examined alongside the chronic deafferentation and recovery animals. All animals in these studies were taken from the same animal population concurrently with experimental animals, and fish lengths, weights, brain weights, and sex were recorded. In control animals, there was a significant increase in body length at 8 weeks and 16 weeks when compared to the length of control animals at day 0 (Figure 4A). Additionally, animal weights significantly increased at 8 and 16 weeks compared to day 0 control animal weights (Figure 4B). There was also a significant increase in brain weight from day 0 to 16 weeks (Figure 4C).

**Figure 5:**
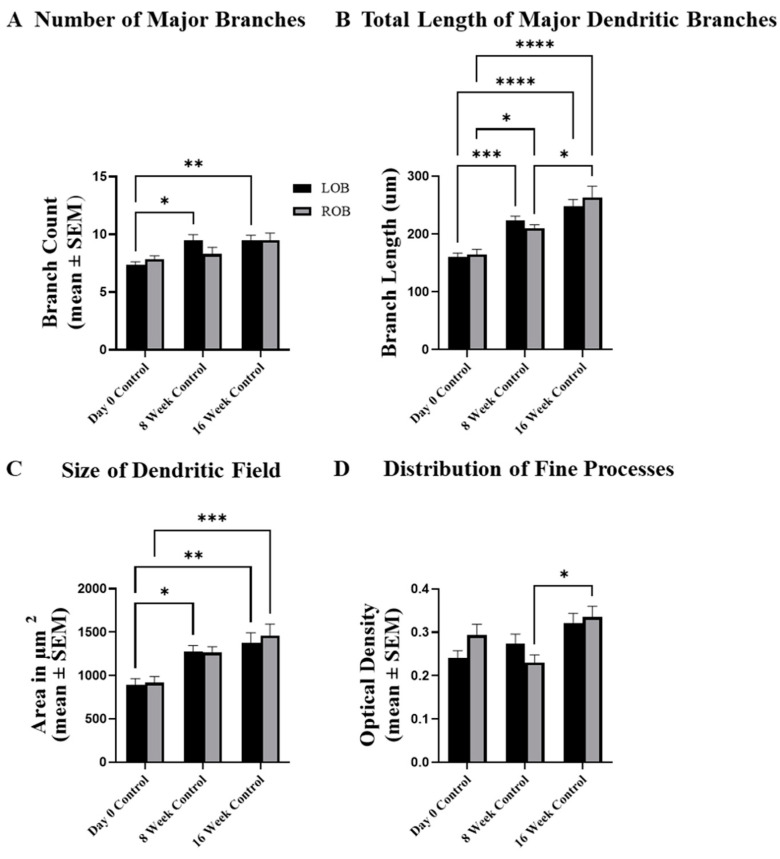
The potential for growth-related changes to dendritic arbor structure was examined in a group of unlesioned cohort control animals over 16 weeks. (**A**) Compared to day 0 control animals, there was a significant increase in the number of major branches in the left olfactory bulbs of animals examined at 8 and 16 weeks. (**B**) At 8 weeks there was a significant increase in the total length of major dendritic branches of mitral cells in the left and right olfactory bulbs compared to day 0 control cells, and this increase was maintained at 16 weeks. Also, at 16 weeks the lengths of major dendritic branches in the right olfactory bulb were significantly longer than those in the right olfactory bulb at 8 weeks. (**C**) There was a significant increase in the relative size of the dendritic field of mitral cells in the left olfactory bulb at 8 weeks compared to day 0 control cells, and at 16 weeks mitral cell dendritic arbors in both the right and left olfactory bulbs were significantly larger than those at day 0. (**D**) The distribution of fine processes in the dendritic arbor remained fairly stable over time, with the only significant increase occurring in cells found in the right olfactory bulb at 16 weeks when compared to those present in the right olfactory bulb at 8 weeks. LOB = left, internal-control olfactory bulb, ROB = right, treated olfactory bulb. For day 0 control, n = 5 fish, 26 mitral cells (LOB), 28 mitral cells (ROB). For 8-week controls, n = 4 fish, 21 mitral cells (LOB), 20 mitral cells (ROB). For 16-week controls, n = 4 fish, 21 mitral cells (LOB), 20 mitral cells (ROB). Two-way ANOVA, with Tukey’s multiple comparisons test, **** = *p* < 0.0001; *** = *p* < 0.001; ** = *p* < 0.01; * = *p* < 0.05. F-value is 10.48 (**A**), 41.38 (**B**), 17.98 (**C**), and 6.12 (**D**). Degrees of freedom is 2, for all. 95% Confidence interval is −0.48 to 0.94 (**A**), −19.78 to 14.61 (**B**), −181.10 to 115.90 (**C**), and −0.04 to 0.03 (**D**).

To examine potential growth-related changes in MC dendrites, the total number of major dendritic branches, the length of those branches, the relative area of the dendritic arbor, and the optical densities of dextran labeling of dendritic tufts of MCs were examined in control fish at day 0, 8 weeks, and 16 weeks. There were no significant differences in the total number of major dendritic branches between the right and left olfactory bulbs in day 0, 8-week, and 16-week control MC dendritic arbors (Figure 5A). There was, however, a significant increase in the total number of major branches in MC dendritic arbors of left olfactory bulbs at 8 and 16 weeks when compared to day 0 controls (Figure 5A). The total length of the major dendritic branches was not significantly different between the left and right olfactory bulbs of day 0, 8-week, and 16-week controls (Figure 5B), but at 8 weeks and 16 weeks the total length of major dendritic branches was significantly increased compared to day 0 control cells. Additionally, the total length of major dendritic branches of MCs in the right olfactory bulb at 16 weeks was significantly increased from those in the right olfactory bulb at 8 weeks, indicating substantial growth-related changes to the dendritic arbor over time (Figure 5B).

The size of the dendritic field of MCs in the left olfactory bulb at 8 weeks, and both olfactory bulbs at 16 weeks, were significantly larger than the size of the dendritic field of MCs examined at day 0 (Figure 5C), although there were no differences in the size of the dendritic field between MCs in the left and right olfactory bulbs at day 0, 8 weeks, or 16 weeks. Optical density of dextran labeling of MC dendritic arbors showed no differences in the estimate of fine processes between left and right bulbs of animals at day 0, 8 weeks, or 16 weeks, but there was a significant increase in the optical density labeling of MC dendritic arbors in the right olfactory bulb at 16 weeks when compared to that in the right olfactory bulb at 8 weeks, indicating a slight change over time in the distribution of fine processes within the dendritic arbors of MCs (Figure 5D).

**Figure 6:**
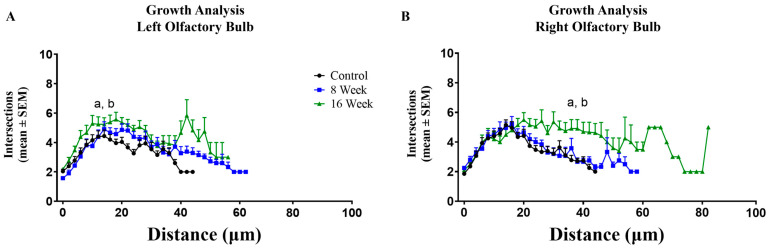
A modified Sholl analysis was used to determine the potential effects of growth on overall dendritic arbor complexity in unlesioned cohort control fish over time. (**A**) In the left olfactory bulb there was a significant increase in overall dendritic complexity at 16 weeks compared to both day 0 (*p* = 0.002) and 8-week (*p* = 0.011) controls. (**B**) In the right olfactory bulb there was a significant increase in overall dendritic complexity at 16 weeks compared to both day 0 (*p* = 0.02) and 8-week (*p* = 0.01) controls. For controls, n = 5 fish, 26 mitral cells (LOB), 28 mitral cells (ROB). For 8-week controls, n = 4 fish, 21 mitral cells (LOB), 20 mitral cells (ROB). For 16-week controls, n = 4 fish, 21 mitral cells (LOB), 20 mitral cells (ROB). Kolmogorov-Smirnov test compared lines formed by the average number of intersections at various distances from the base of the arbor, a = *p* < 0.05 compared to day 0 control cells, b = *p* < 0.05 compared to 8-week control cells.

The overall dendritic complexity of MCs of untreated, cohort control animals was analyzed at day 0, 8 weeks, and 16 weeks using a modified Sholl analysis. Kolmogorov-Smirnov tests indicated that the dendritic arbors of MCs in the left and right olfactory bulbs of animals at 16 weeks were significantly more complex than MC dendritic arbors at both day 0 and 8-week timepoints, indicating that MC dendritic arbors become more complex with growth over time (Figure 6A,B).

### 2.3. Injury and Recovery Normalized to Growth-Related Changes

**Figure 7:**
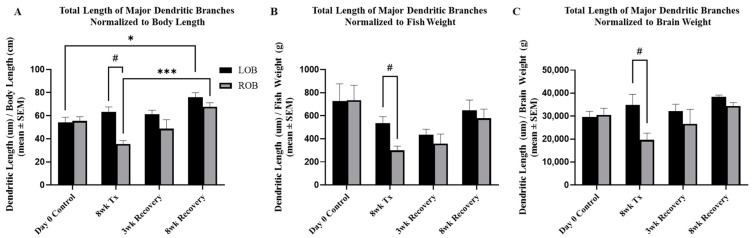
Total length of major dendritic branches normalized to body length, fish weight and brain weight following 8 weeks deafferentation and 3 weeks or 8 weeks of recovery. (**A**) After normalizing total length of major dendritic branches to fish body length, the treated right olfactory bulbs were still significantly different following 8 weeks of chronic, partial deafferentation compared to the internal control left olfactory bulb. Following 8 weeks of recovery, normalized data showed the left and right olfactory bulbs were increased significantly compared to day 0 controls and the 8-week injury timepoint, respectively. (**B**) When normalized to fish weight, total length of dendritic branches in the treated right olfactory bulbs were still significantly different following 8 weeks of chronic, partial deafferentation compared to the internal control left olfactory bulb. (**C**) Normalizing total length of major dendritic branches to brain weight still showed a significant difference in the treated right olfactory bulbs following 8 weeks of chronic, partial deafferentation compared to the internal control left olfactory bulb. LOB = left, internal-control olfactory bulb, ROB = right, treated olfactory bulb. Paired t-test compared to internal controls, # = *p* < 0.05; Two-way ANOVA, with Tukey’s multiple comparisons test, *** = *p* < 0.001; * = *p* < 0.05. F-value is 10.54 (**A**), 5.22 (**B**), and 3.15 (**C**). Degrees of freedom is 3, for all. 95% Confidence interval is 5.54 to 18.26 (**A**), −51.39 to 237.20 (**B**), and 1327 to 10,596 (**C**).

To distinguish whether recovery of MC dendritic morphology was a measure of regeneration as opposed to an artifact of growth-related changes, the data were normalized to individual values for the physical characteristics of body length, fish weight and brain weight. As shown in Figure 4, these parameters increased significantly with age. MC total dendritic branch length retained significance at the 8-week chronic deafferentation timepoint when normalized to body length, fish weight and brain weight (Figure 7A–C), suggesting that regeneration of branch length could potentially function relatively independently of growth-related changes. Additionally, normalization of the data revealed significant increases in the left olfactory bulb at the 8-week recovery timepoint compared to the day 0 control and in the right olfactory bulb compared to the 8-week deafferentation timepoint (Figure 7A). For all other MC measures when normalized to body length, fish weight and brain weight, the number of major branches, the area of the dendritic arbor and measures of optical density were no longer significant at the 8-week chronic deafferentation timepoint, suggesting that recovery of these characteristics could be a function of growth.

## 3. Discussion

Plasticity of synaptic connections is essential for the development and maintenance of dendritic shape, which is critical for proper neural functioning [26,27,28]. Research involving the effects of afferent activity on dendrites has been conducted primarily in developmental systems [29,30]. Fewer studies have been performed in adult animals, and most of those examine the plasticity and response to deafferentation of dendritic spines [31,32,33]. Some researchers have taken advantage of the fact that the olfactory bulb is an adult brain structure that is continually developing, so developmental processes can be examined in an adult animal. Mizrahi [34] investigated dendritic development in adult-born periglomerular neurons. Among his findings was that the dendrites of aspinous periglomerular neurons are more dynamic, while spinous periglomerular and granule neurons show stability in the dendritic branches and plasticity in the spines. His group also examined mitral/tufted cell dendrites and found that they too are stable in adult mice, even with increased activity through odor training or with pharmacology [35]. Another group examined adult-born granule cells in olfactory bulb slices and found that the dendrites of these spinous neurons are more dynamic in early stages of formation but showed less filopodial activity as they mature [23].

In general, there is a lack of information on the effects of deafferentation and recovery on aspinous dendrites in adult brain structures, and we set out to fill in this gap using a popular model animal. Additionally, while it is known that adult zebrafish maintained in a constant, optimal temperature environment exhibit continued growth with age [25], we found no studies examining the role of growth on dendritic arbor structure and complexity. The fact that our study included long treatment times in an indeterminate grower allowed us also to examine effects of growth in adults. The purpose of this study was to examine the potential recovery of MC dendritic arbor structures that are reduced by chronic partial deafferentation and observe any potential growth-related changes to adult MC dendritic arbors over time.

Our model of reversible deafferentation allows us to investigate both degeneration and regeneration in the olfactory bulb of adult zebrafish. Chronic, partial deafferentation of the olfactory bulb results in severe alterations to MC dendritic arbor morphology [24]. It is known that some mitral cells project their axons through the anterior commissure to a similar location in the contralateral olfactory bulb [36]. Thus, in the current study, comparisons were made both to internal control olfactory bulbs and to the olfactory bulbs of untreated, external control fish. Following 8 weeks of deafferentation, MCs retain some of their major dendritic branches; however, smaller secondary branches are noticeably diminished, and the fine processes that establish the dendritic tuft of control cells are notably lacking in deafferented MCs. The morphology of deafferented MCs is in harsh contrast to the control morphology of unidendritic cell arbors, which maintain normal dendritic arbor characteristics. We show here that adult zebrafish MCs can recover their dendritic structure when afferent input is restored. When given time to recover for 3 weeks following deafferentation, dendritic arbors continued to lack fine processes and smaller branches, but appeared to be in a transitory state of returning to control levels. Following 8 weeks of recovery, MC dendritic morphology regained robust fine processes as well as control levels of primary and major branches. When allowed 3 weeks of recovery, the number of major branches, the distribution of fine processes and overall dendritic complexity were no longer significantly decreased. This indicates that the process of recovering dendritic structures occurs much more quickly than the process of losing them. When allowed 8 weeks to recover from chronic, partial deafferentation there were no differences between previously deafferented MCs in general morphology or in dendritic arbor quantifications compared to internal control cells; however, both were significantly increased from day 0 unlesioned control cells. Increased dendritic complexity in developing hippocampal neurons is activity dependent and uses a Semaphorin 3A pathway [37]. Relatedly, adult-born granule cells of the olfactory bulb show increased dendrite dynamics in response to activity as they are developing, but they do not require activity for filopodia extension when mature [23]. Interestingly, Mizrahi [34] found that sensory deprivation from naris occlusion in mice had no effect on dendrite complexity, at least during development of adult-born periglomerular cells. Our report of recovery of dendritic arborization following return of afferent input is consistent with some of these findings, and the fact that activity is not necessary in some instances is likely due to developmental stage of the neurons, where our study focused on mature mitral cells of the adult olfactory bulb.

The discovery of differences between day 0 control cells and internal control cells at recovery time periods led to the examination of potential growth-related changes to MC dendritic arbors. Unlesioned control fish were examined over time, and results revealed that over the course of a 16-week long experiment there were significant increases in animal length, weight, and brain weight. These changes may correspond to the significant increases in the number of major branches, the total length of those major branches, and the size of the dendritic field of MCs. Most notably, there were significant increases in the overall complexity of dendritic arbors at 16 weeks when compared to both day 0 and 8-week control arbors indicating that there was continued growth and elaboration of MC dendrites.

Many molecules involved in dendrite morphogenesis have been identified across different model systems and locations [38,39]; however, most of these have been shown in culture or in developing animals [18,40,41]. The process by which dendritic arbors elaborate and grow in proportion to animal growth during development is referred to as dendritic scaling [36], and this may continue throughout life. Growth and elaboration of dendritic arbors in correlation to animal size has been rarely studied in adult animals, although examples do exist. Goldfish demonstrate continued growth of dendritic arbors in retinal ganglion cells [42,43]. Examination of pyramidal neurons in the prefrontal cortex and basolateral amygdala of rats growing from juvenile to pubescent stages show an increase in overall dendritic length, complexity and spine density; growth from puberty into adulthood results in decreased dendritic spines and increased number of dendritic branches [44]. Many questions remain as to how and why dendrites coordinate and regulate adult arbor structures. It is interesting to find that the normalized data for MC total dendritic branch length, when considering individual growth-related physical characteristics, uniquely measures regenerative plasticity in the zebrafish olfactory bulb. For other measures of dendritic morphology, further experimentation may be required to elucidate whether the changes in complexity are functions of regenerative or growth-related plasticity. We report here that mitral cells in the adult zebrafish olfactory bulb recover from deafferentation-induced loss of dendritic branches and that fish growth likely influences the dendrite recovery process. Regardless of whether the restoration of dendritic arbors results from normal, age-related growth or dendritic regeneration, the capacity for the restoration of adult dendritic arbors to baseline morphology is novel, as this has not been previously shown in other studies. This study provides evidence of the plasticity of adult dendritic arbor structures in a complex model organism and further proves that the zebrafish is a superb model organism for understanding neuroregeneration.

## 4. Materials and Methods

### 4.1. Animals

A total of 19 adult male and 19 adult female zebrafish, *Danio rerio*, over 6 months of age were used, and 276 mitral cells were examined. The fish were maintained in 15-gallon aquaria filled with conditioned fish water (reverse osmosis water treated with dechlorinating solutions and conditioning salts) at 28.5 °C and were fed twice daily (morning and afternoon) with commercial flake food (Tetra) under natural lighting conditions. Characteristics including sex, length, weight, and brain weight were collected at the conclusion of the experiment. Fish were obtained from local commercial sources and all animal care protocols and experimental procedures were approved by the Institutional Animal Care and Use Committee.

### 4.2. Chronic Deafferentation

Chronic, partial deafferentation of the olfactory bulb was achieved via repeated chemical lesioning of the olfactory epithelium with detergent as described previously [7,24]. Zebrafish were anesthetized with 0.03% MS222 (3-amino benzoic acid ethyl ester, Sigma-Aldrich, St. Louis, MO, USA) in fish water. After anesthesia was confirmed with caudal fin pinch, fish were placed on a clay dish and a pulled Wiretrol capillary pipette tip was used to lavage the right nasal cavity with approximately 1 µL of 0.7% Triton X-100 (Sigma-Aldrich, St. Louis, MO, USA) and 0.005% methylene blue in 0.1 M phosphate-buffered saline, leaving the left olfactory epithelium to serve as the untreated internal control. The Triton X-100 solution was kept in contact with the tissue for two minutes. This procedure was repeated once every three days for eight weeks after which fish were euthanized or allowed to recover for three or eight weeks. Cohort control fish were euthanized along with the deafferented fish, and day 0 control fish were euthanized on the first day of the experiment.

### 4.3. Olfactory Tract Tracing

MCs from deafferented and control fish were labeled using retrograde tract tracing from the olfactory tracts [12,24]. For this technique, fish were over-anesthetized with 0.03% MS222 and then perfused with pH 7.4 phosphate-buffered saline before immediate brain dissection under a stereomicroscope. Approximately 0.05–0.1 µL of Texas Red Dextran (10,000 MW, 5 mg/mL in PBS, ThermoFisher Scientific, Waltham, MA, USA, catalog # D1863) was injected into both the medial and lateral olfactory tracts within the telencephalon. Following injection, brains were incubated at 28.5 °C in artificial fish cerebrospinal fluid [100 mM NaCl, 2.46 mM KCl, 1 mM MgCl_2_H_2_O, 0.44 mM NaH_2_PO_4_H_2_O, 1.13 mM CaCl_2_H_2_O, 5 mM NaHCO_3_; [45]] with 3–5% CO_2_ for approximately four hours [12,24]. Whole-brain specimens were then fixed in 4% paraformaldehyde for 24 h at 4 °C, rinsed in buffer, mounted between two coverslips, and viewed on a Nikon C2 confocal microscope.

### 4.4. Dendritic Analysis

Unidendritic MCs are the most predominant and uniform cell type within the zebrafish olfactory bulb [12]. For dendritic analysis, a minimum of five unidendritic cells were selected randomly from each right and left olfactory bulb from at least four fish from each treatment or control group. Confocal microscopy with z-stack imaging was used to gather fine optical sections of tissue throughout the olfactory bulb at 0.25 µm intervals. Cellular profiles were used to create two-dimensional projection images analyzed with Fiji, an open platform for biological image analysis [46]. Utilizing the Simple Neurite Tracer Plugin, quantification of major dendritic branches, total length of dendritic branches, size of dendritic field, optical density measures of fine processes of the dendritic arbor, and modified Sholl analysis were performed by tracing the two-dimensional outline of the arbor of the major primary, secondary, tertiary, and quaternary branches that possessed clearly defined borders. Optical density was measured with Fiji by converting the mean gray level from the area of the arbor using the formula OD = −log (intensity of background/intensity of area of interest). Examination of overall complexity was conducted using a modified Sholl analysis, where the concentric circles originated at the base of the dendritic arbor [24]. Data from cells from right and left olfactory bulbs were compared within groups using paired, two-tailed t-tests and between groups using ANOVA with Tukey’s test for multiple comparisons. Data from the Sholl analysis was analyzed using paired t-tests to compare the number of intersections at each distance from the base of the arbor, and a Kolmogorov-Smirnov test was performed to determine significant differences in the lines formed by the number of intersections at each distance from the base of deafferented and control dendritic arbors; *p* values less than 0.05 were considered significant.

## Data Availability

Dataset available on request from the authors.
